# Does the Attentional Boost Effect Depend on the Intentionality of Encoding? Investigating the Mechanisms Underlying Memory for Visual Objects Presented at Behaviorally Relevant Moments in Time

**DOI:** 10.3389/fpsyg.2020.584187

**Published:** 2020-11-09

**Authors:** Fabian Hutmacher, Christof Kuhbandner

**Affiliations:** ^1^Department of Psychology, University of Regensburg, Regensburg, Germany; ^2^Human-Computer-Media Institute, University of Würzburg, Würzburg, Germany

**Keywords:** attentional boost effect, visual long-term memory, incidental encoding, intentional encoding, perceptual long-term memory

## Abstract

Pictures in a rapid serial visual presentation (RSVP) stream are better remembered when they are simultaneously presented with targets of an unrelated detection task than when they are presented with distractors. However, it is unclear whether this so-called “attentional boost effect” depends on the intentionality of encoding. While there are studies suggesting that the attentional boost effect even occurs when encoding is incidental, there are several methodological issues with these studies, which may have undermined the incidental encoding instructions. The present study (*N* = 141) investigated the role of the intentionality of encoding with an improved experimental design. Specifically, to prevent a spill-over of intentional resources to the pictures in the RSVP stream, the speed of the stream was increased (to four pictures per second) and each picture was presented only once during the course of the experiment. An attentional boost effect was only found when encoding was intentional but not when encoding was incidental. Interestingly, memory performance for incidentally encoded pictures was nevertheless substantially above chance, independently of whether images were presented with search-relevant targets or distractors. These results suggest that the attentional boost effect is a memory advantage that occurs only under intentional encoding conditions, and that perceptual long-term memory representations are formed as a natural product of perception, independently of the presence of behaviorally relevant events.

## Introduction

Based on the observation that only a fraction of our visual field is represented in high resolution, while non-foveated, peripheral information is represented in reduced fidelity (see, e.g., Rosenholtz, 2011; Cohen et al., 2016) as well as based on phenomena such as change blindness (e.g., Rensink et al., 1997) or inattentional amnesia (e.g., Simons and Chabris, 1999), it has become a widely accepted idea that most perceptual information is rapidly forgotten or never stored at all (for a critical review of this perspective, see, e.g., Dudai, 1997; Brady et al., 2011; Hutmacher, 2020). However, this assumption has been challenged by various studies published in the last decade, demonstrating that detailed and durable long-term memory representations are formed as a natural product of perception. While most studies have investigated visual long-term memory (Vogt and Magnussen, 2007; Brady et al., 2008; Konkle et al., 2010; Kuhbandner et al., 2017), similar results have been obtained for auditory (Hutmacher and Kuhbandner, 2020) and haptic (Hutmacher and Kuhbandner, 2018) long-term memory (for the reasons behind the dominance of vision in research, see Hutmacher, 2019). In short, performance in these studies indicated that much more of the incoming perceptual information is stored in long-term memory than previously believed. However, the participants’ memory was not perfect, that is, the participants did not remember *all* of the presented stimuli. Thus, an interesting question remains: What are the mechanisms that select whether a stimulus is stored in long-term memory?

One possible answer to this question is provided by the idea that the encoding of perceptual information is enhanced at behaviorally relevant moments in time: When something important happens in our environment, it seems adaptive to store not only the stimulus to which we react but also the seemingly unrelated surroundings. The existence of such a selection mechanism is demonstrated by the so-called “attentional boost effect” (for a review, see Swallow and Jiang, 2013). In the typical attentional boost paradigm, participants perform two concurrent but unrelated tasks. One task is to view a series of pictures and to remember them for a later memory test. The other, concurrently executed task is to press a button when a target (e.g., a white square) appears in a series of distractors (e.g., a black square). Typically, the targets and distractors are superimposed on the pictures. Although the two tasks are completely unrelated, memory performance for pictures that are paired with a target in the concurrent task are remembered better in a subsequent memory test than those that are paired with a distractor (see, e.g., Lin et al., 2010; Swallow and Jiang, 2010, 2011, 2012, 2014a; Leclercq and Seitz, 2012a,c; Leclercq et al., 2014b). The attentional boost effect has been replicated under different levels of uncertainty (Leclercq et al., 2014a), using pupillometry (Hoffing and Seitz, 2015) and implicit memory tests (Spataro et al., 2013), as well as for verbal material (Mulligan et al., 2014; Protopapas et al., 2017; but see Walker et al., 2017) and emotional stimuli (Rossi-Arnaud et al., 2018).

As described above, the standard procedure for investigating the attentional boost effect is based on a dual-task paradigm in which both streams of information are relevant for the participants: They are asked to detect target squares *and* to remember the pictures presented in the background for a later memory test. Thus, a crucial question arises: Does the presentation of the target lead to a general enhancement in perceptual processing, as hypothesized by Swallow and Jiang (2013, 2014a), or is this advantage limited to settings in which the visual stimuli in the background are encoded intentionally?

A straightforward way of answering this question is to compare performance when participants are instructed to remember the pictures in the background for a later memory test (*intentional encoding*) with performance when participants are instructed to ignore the pictures in the background, as they are irrelevant to the current task (*incidental encoding*). In fact, the question whether the attentional boost effect can also be found when encoding is incidental has been investigated in several studies, albeit with mixed results. While some studies (Dewald et al., 2011; Swallow and Jiang, 2011; Leclercq and Seitz, 2012b, Experiment 4) found no attentional boost effect when encoding was incidental, other studies did (Dewald et al., 2013; Swallow and Jiang, 2014b; Broitman and Swallow, 2019, Experiments 2 and 3). It has consequently been argued that the attentional boost effect can occur when encoding is incidental, although the magnitude of the effect may be reduced under such conditions (see Swallow and Jiang, 2014b; see Choi et al., 2009 as well as Tsushima et al., 2008, for possible explanations).

Before accepting this conclusion, however, it seems important to take a closer look at the way the intentionality of encoding was manipulated in the studies that found an attentional boost effect under incidental encoding instructions. As described above, participants were instructed to ignore the pictures in the background to ensure that encoding is incidental. Although this is likely to rule out intentional memorization strategies in preparation for a later memory test, the overall effectiveness of such an instruction also depends on the specific characteristics of the task. For instance, when the demands in the detection task are relatively low, the remaining attentional resources may spill over to task-irrelevant items (see, e.g., Lavie, 1995, 2010). In particular, participants may choose to encode the background pictures although they have been deemed irrelevant when performing the detection task is not experienced as challenging enough or when the background pictures attract their attention.

In fact, this may potentially have been the case in the studies that found an attentional boost effect under incidental encoding instructions for several reasons. First, in all of these studies, the same pictures were presented several times, ranging from three (Swallow and Jiang, 2014b) to eight (Broitman and Swallow, 2019) and 120 times (Dewald et al., 2013).^1^ Second, the pictures were presented at a rate of one picture every 500 ms (i.e., with an SOA of 500 ms). As it takes no longer than about 150 ms to process even a complex natural image (Thorpe et al., 1996), and as the concurrent detection task requires relatively simple decisions, one could hypothesize that the remaining time and attentional resources were used to encode the pictures. Third, while the pictures in the background were visible for 500 ms, the search target and distractor stimuli were presented for only 100 ms in two of the three aforementioned studies (Swallow and Jiang, 2014b; Broitman and Swallow, 2019). In other words, the supposedly irrelevant pictures were visible on the screen for further 400 ms after the relevant target had already disappeared, leaving ample room for encoding.

In short, while encoding was incidental in these studies in the sense that participants did not know that their memory for the background images would be tested later, encoding may still have been intentional in the sense that participants may have chosen to encode the background stimuli for various reasons, as they had sufficient time and attentional resources for doing so. The present study was set up to account for this possibility, and to provide a clear test for determining whether the attentional boost effect depends on the intentionality of encoding.

Specifically, compared to the previous studies that found an attentional boost effect under incidental encoding instructions, we made three adjustments. First, each picture (the image of an everyday object) was presented only once during the course of the experiment. Second, the presentation speed of the pictures was increased (to four pictures per second, i.e., an SOA of 250 ms). Third, the search target and distractor stimuli (squares) in the foreground were presented for the same amount of time as the pictures. To examine the role of the intentionality of encoding, encoding was incidental for half of the participants and intentional for the other half. If an attentional boost effect occurs in both conditions, the attentional boost effect would stem from a general enhancement in perceptual processing. If no attentional boost effect occurs in the incidental encoding condition, the attentional boost effect should better be viewed as a memory advantage that occurs only when stimuli are encoded intentionally.

## Materials and Methods

### Participants

We decided to collect data from at least 27 participants per group in order to have sufficient power (0.95, alpha = 0.05, two-tailed) to detect medium sized effects in a between-subjects design (*f* = 0.25; G*Power 3.1.9.7, Faul et al., 2007), and to continue data collection until the end of the semester. In total, we recruited 143 undergraduate students. Due to a computer crash, two participants could not finish the experiment. Thus, the data of 141 participants (106 female, 34 male, 1 diverse; age: *M* = 20.94 years, *SD* = 1.73, 18–29 years) were included in the analysis. Half of them (*N* = 70) performed the experiment under incidental encoding instructions, the other half (*N* = 71) under intentional encoding instructions. Participants received five euros and an additional amount of money based on their performance (see below for details). All participants provided written informed consent and reported normal or corrected-to-normal vision. During recruitment, potential participants were asked not to take part in the study when suffering from defective color vision. The study was conducted in accordance with the Helsinki Declaration and the University Research Ethics Standards. In Germany, these types of psychological studies do not require ethical approval of an Ethics Committee.^2^ All data exclusions, manipulations, and measures in the experiment are reported. Data can be downloaded at https://osf.io/6fej2/.

### Apparatus

The stimuli were presented on a 23 inch LG 23ET63V monitor with a resolution of 1,920 by 1,080 pixel and a vertical refresh rate of 60 Hz. Viewing distance was about 50 cm. The experiment was programmed using the E-Prime 2.0 software (Psychology Software Tools, Inc., 2012). Participants sat unconstrained in a normally lit interior room. Room lighting was kept constant by closing the window shutters.

### Materials

Prior to the experiment, 840 pictures of everyday objects were randomly chosen from a database containing pictures of 2,400 unique objects (Brady et al., 2008). The same 840 pictures were used for all participants.

A fraction of these pictures was used as *filler objects* to separate trials during the detection task (*n* = 240). The filler objects were the same across participants. As memory for the pictures was tested using a two-alternative-forced-choice recognition test (2AFC; see below), the remaining pictures were divided into two picture sets, which served either as *old objects* (shown in the detection task; *n* = 300) or *new objects* (not shown in the detection task; *n* = 300) in the recognition test. Which of the picture sets served as old and new objects was counterbalanced across participants. During the detection task, two colored squares (pink and green) were used as targets and distractors, respectively. Whether the pink or the green square was the target square was counterbalanced across participants. The colors were chosen so that the squares were clearly distinguishable from the objects presented in the background.

### Design and Procedure

**Figure 1:**
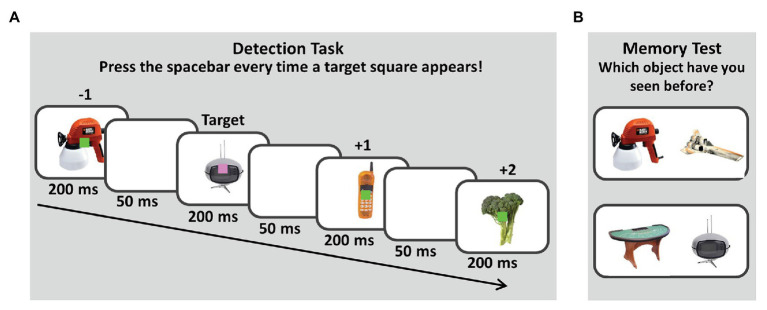
Memory paradigm. The experiment consisted of two phases. In an initial detection task depicted in **(A)** participants viewed a rapid stream of pictures presented at the center of the screen, overlapped by a colored square. Participants were asked to press the spacebar as quickly as possible whenever they saw a target square (here: pink) and to make no response whenever the distractor square appeared (here: green). Half of the participants knew that their memory for the pictures would be tested later (*intentional encoding*), while the other half of the participants was asked to ignore the pictures as good as possible (*incidental encoding*). After completing the detection task, participants performed a two-alternative-forced-choice recognition test, depicted in **(B)**. On each trial, a previously presented picture was paired with a new picture. Participants were asked to indicate which of the two pictures they had seen before by pressing one of two keys.

Following the typical paradigm of studies on the attentional boost effect (see, e.g., Swallow and Jiang, 2010), the experiment consisted of two parts: a detection task and a recognition test. During the initial detection task, participants viewed a rapid stream of pictures (8.5° × 8.5°) presented at the center of the screen and overlapped by a colored square (0.9° × 0.9°; for an illustration of the trial procedure, see Figure 1A). Both the picture and the square were shown for 200 ms, followed by a 50 ms blank interstimulus interval. Participants were asked to press the spacebar as quickly as possible whenever they saw a target square and to make no response whenever the distractor square appeared. As the squares and pictures were visible for 200 ms only, it was difficult for participants to press the spacebar, while the target square was still visible on the screen. Thus, participants were instructed to press the spacebar whenever they had seen a target square, even if it had already been replaced by the next trial. In total, 30 target squares were presented during the detection task.

In order to examine the attentional boost effect, serial positions in the detection task have to be fixed. Thus, trials were grouped into blocks of 10 trials. A block of trials started with the presentation of two pictures paired with a distractor square (serial positions -2 and -1, relative to the target), followed by the presentation of a picture paired with a target square (serial position 0) and the presentation of seven pictures paired with a distractor square (serial positions +1 to +7, relative to the target). Each picture was presented only once in the detection task. In which serial position a picture was presented was counterbalanced across participants. To minimize potential effects of temporal regularity, zero to eight filler pictures (all presented with the distractor square) separated the blocks of 10 trials, following the procedure by Swallow and Jiang (2010). The number of filler pictures (zero to eight) that was presented between two blocks of trials was determined randomly after each block of trials.

The crucial manipulation in the present experiment was the way participants were instructed. In the *incidental encoding condition*, participants were asked to ignore the pictures as good as possible. Participants were told that the pictures are irrelevant to the task and that the experiment was designed to investigate how well humans can ignore irrelevant information while performing another task. No mention was made that memory for any of the pictures would be tested later. In the *intentional encoding condition*, we followed the instructions typically used in studies on the attentional boost effect. That is, participants were instructed to remember the pictures presented during the detection task for a later memory test. The exact nature of the memory test was not explained.

After completing the detection task, participants performed a 2AFC recognition test (for an illustration, see Figure 1B). On each trial, an old picture, which had been presented in the detection task was paired with a new picture. Participants were asked to indicate which of the two pictures they had seen before by pressing one of two keys. Participants were asked to follow their “gut feelings” when not knowing the answer and proceeded at their own pace. Participants received feedback whether their response was correct or incorrect (750 ms). For each correct answer, participants received 5 cents. For each wrong answer, 5 cents were subtracted. The total amount of money participants received in addition to the fixed amount of 5 euros was shown on the screen after completing the experiment. Except from the filler pictures, all pictures from the detection task were tested in the 2AFC recognition test (300 memory test trials). Whether the novel picture or the previously presented old picture was shown on the left or on the right was counterbalanced within participants. The order of testing was random.

## Results

### Detection Task

The first key press after the presentation of a target was counted as a correct response as long as it was made during the same block of trials. Participants reliably detected the target square, both when encoding was incidental (*M_Accuracy_* = 89.48%, *SD* = 10.27) and when encoding was intentional (*M_Accuracy_* = 83.43%, *SD* = 10.88). Target detection performance was significantly better under incidental encoding instructions, *t*(139) = 3.39, *p* = 0.001, *d* = 0.57. In addition, reaction times were lower under incidental encoding instructions (*M_incidental_* = 384 ms, *SD* = 63; *M_intentional_* = 412 ms, *SD* = 70), *t*(139) = 2.52, *p* = 0.013, *d* = 0.42. The distribution of the key presses across the serial positions in response to the target was highly similar across the two conditions. Most responses were either given while the target was still present (i.e., at serial position 0; incidental: 10.43%, intentional: 8.16%) or one trial after the target had disappeared (i.e., serial position 1; incidental: 83.08%, intentional: 82.22%). Some responses were also given two trials after the target had disappeared (i.e., serial position 2; incidental: 3.99%, intentional: 5.40%), while later responses (i.e., serial positions 3–7) were extremely rare (incidental: 2.50%, intentional: 4.22%).

### Memory Performance

**Figure 2:**
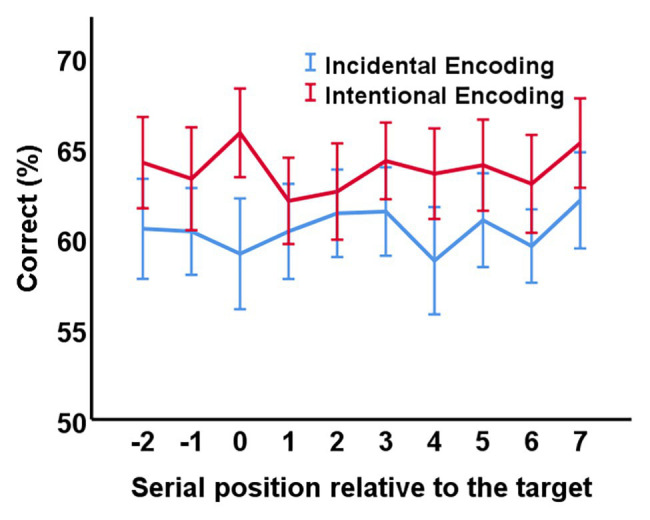
Results. The percentage of correctly remembered pictures is shown as a function of condition (*incidental encoding* vs. *intentional encoding*) and serial position (−2 to +7). Serial position 0 represents the presentation of a target square. Error bars represent 95% confidence intervals.

A detailed depiction of the memory performance for the pictures presented at the different serial positions can be found in Figure 2. For the statistical analysis, the nine non-target positions (-2 and -1 as well as +1 to +7) were integrated into one estimate (see e.g., Swallow and Jiang, 2010). Next, we ran a 2 × 2 ANOVA with the between-subjects factor instruction (*incidental* vs. *intentional encoding*) and the within-subjects factor position (*target* vs. *non-target*). The main effect for instruction was significant, *F*(1,139) = 12.24, *p* = 0.001, *η*^2^ = 0.08, indicating that overall memory performance was better when the pictures were encoded intentionally (*M* = 63.81%, *SD* = 6.58) than when they were encoded incidentally (*M* = 60.47%, *SD* = 6.97). The main effect for position was not significant, *F*(1,139) = 0.22, *p* = 0.642, *η*^2^ = 0.002. However, there was a significant instruction by position interaction, *F*(1,139) = 5.16, *p* = 0.025, *η*^2^ = 0.04.

In order to better understand the significant interaction, we took a closer look at performance in the different conditions (see Table 1). When encoding was intentional, performance for the target position was better than performance for the non-target positions, that is, there was an attentional boost effect [*M_Difference_* = 2.24%, *SD* = 9.34, 95% CI (0.03; 4.45)]. However, when encoding was incidental, performance was numerically worse for the target position than for the non-target positions, that is, there was no attentional boost effect [*M_Difference_* = -1.48%, *SD* = 10.08, 95% CI (-3.88; 0.93)]. In addition, participants performed better when encoding was intentional than when encoding was incidental, both for the target position [*M_Difference_* = 6.68%, *SD* = 11.65, 95% CI (2.80; 10.56)], and the non-target positions [*M_Difference_* = 2.97%, *SD* = 6.74, 95% CI (0.72; 5.21)].

**Table 1:** Performance in the different conditions.

	Target		Non-target	
	*M* (%)	*SD*	*M* (%)	*SD*
Intentional encoding	65.82	10.34	63.58	6.71
Incidental encoding	59.14	12.84	60.62	6.77

## Discussion

The present study was designed to answer the question whether the attentional boost effect depends on the intentionality of encoding. Previous research has reported mixed results (Dewald et al., 2011, 2013; Swallow and Jiang, 2011, 2014b; Leclercq and Seitz, 2012b; Broitman and Swallow, 2019). However, a closer look at the studies that found an attentional boost effect under incidental encoding instructions indicates that encoding may not have been completely incidental as the to-be-ignored pictures were presented several times with a relatively long presentation duration. In the present study, to ensure that encoding was truly incidental, presentation speed was increased to four pictures per second and each picture was presented only once during the course of the detection task. Under such conditions, an attentional boost effect was only found when encoding was intentional but not when encoding was incidental.

Performance in the target detection task indicated that the intentionality of encoding was manipulated successfully. Participants’ target detection performance was lower and their reaction time was longer when they were instructed to remember the pictures in the background in addition to searching for presented targets, compared to when they were instructed to ignore the background pictures. That is, participants followed the instructions and paid more attention to the target detection task in the incidental encoding condition compared to the intentional encoding condition. This was also supported by the finding that overall memory performance for the pictures was worse in the incidental encoding condition compared to the intentional encoding condition, replicating the finding that the intention to memorize new information enhances recognition memory (e.g., Neill et al., 1990). In sum, these findings suggest that the methodological adjustments made in the present study have helped to ensure that performance in the incidental encoding condition was not driven by uncontrolled encoding strategies.

In three previous studies, an attentional boost effect was reported even when participants were instructed to focus on the target detection task and to ignore the pictures presented in the background (Dewald et al., 2013; Swallow and Jiang, 2014b; Broitman and Swallow, 2019). However, in these studies, the to-be-ignored pictures were presented several times and for a substantially longer amount of time than necessary to detect the target. Under these conditions, attentional resources may have spilled over to the to-be-ignored pictures (e.g., Lavie, 1995, 2010), which may have undermined the incidental encoding instructions. The fact that no attentional boost effect occurs under incidental encoding instructions when encoding is completely incidental, challenges the assumption that the attentional boost effect mirrors a general enhancement in perceptual processing (see Swallow and Jiang, 2013, 2014a). Rather, it seems that the attentional boost effect is a processing advantage that occurs only when participants try to memorize the background pictures intentionally. In fact, such a finding corroborates the results from other studies that have found no attentional boost effect under incidental encoding instructions (Dewald et al., 2011; Swallow and Jiang, 2011; Leclercq and Seitz, 2012b). Nevertheless, an independent replication of the present findings by other research groups seems desirable.

The present study revealed another intriguing finding: Despite the fact that the pictures were presented very rapidly (four pictures per second) and each of the pictures was presented only once during the course of the detection task, performance was far above chance, even when participants were instructed to ignore the pictures as good as possible. This fits well with several recent studies demonstrating that perceptual long-term memory representations are formed as a natural product of perception, independently of the focus of attention and intention of memorization (e.g., Kuhbandner et al., 2017; Hutmacher and Kuhbandner, 2018, 2020). How astounding this ability actually is, can be illustrated by taking a closer look at the data of the present experiment. In the incidental encoding condition, the observed percentage of correct memory responses was 60.47%. To determine the true percentage of pictures stored in memory (PR_True_), the observed percentage correct (PC_Observed_) has to be corrected for fortunate guesses in a 2AFC (formula: PR_True_ = 2 * PC_Observed_ − 100; see, e.g., Brady et al., 2013), revealing that 20.94% of the pictures were stored in memory in the incidental encoding condition. In effect, this means that about one picture per second was successfully stored in long-term memory – despite the fact that each picture was shown only once for a quarter of a second, and that participants completely focused on the detection task while trying to ignore the pictures as good as possible.

How can this finding be explained? As the present study was not meant to answer this question, future research is needed to unravel the mechanisms underlying performance in the incidental encoding condition. However, one may speculate that a significant fraction of the irrelevant and ignored information is stored simply because it fits with the operating characteristics of human perception and memory. For instance, proponents of predictive coding accounts argue that our current model of the world is constantly refined based on the interplay of sensory inputs and top-down expectations (see, e.g., Friston, 2010; Clark, 2013; Hohwy, 2013). Importantly, this interplay takes place on different hierarchical levels from low-level perception to higher-order cognition. Hence, one could hypothesize that the instruction to focus on the detection task and to ignore the pictures as good as possible changed higher-order cognitive processes such as the intention to allocate the attention on the detection task, but left low-level processes comparably unchanged, enabling the participants to store a certain amount of information and to retrieve it at the later memory test. Such a perspective fits well with models of long-term memory claiming that incoming information can be processed in multiple independently operating, but also interacting subsystems and that even information we are completely unware of can be stored in memory and influence our behavior (Johnson, 1983, 2007; Johnson and Hirst, 1993). In fact, recent studies have shown that high-fidelity long-term memory representations are even formed for unattended, irrelevant, and incidentally encoded information (Kuhbandner et al., 2017; Hutmacher and Kuhbandner, 2020).

The methodological adjustments that were made in the present study compared to the previous studies that had found an attentional boost effect under incidental encoding instructions (i.e., presenting each picture only once during the detection task, increasing the presentation speed to four pictures per second, and presenting the search target and distractor stimuli in the foreground for the same amount of time as the pictures in the background) served a common goal: ensuring that encoding was truly incidental under incidental encoding instructions. In other words, the *combination* of these methodological adjustments was a necessary precondition for being able to differentiate between intentional and incidental encoding. Thus, investigating the impact of each adjustment (or a certain combination of adjustments) on the size of the attentional boost effect did not fall into the scope of the present study. Nevertheless, setting up experiments that systematically investigate the impact of various factors (such as the presentation duration or the number of times a certain picture is shown during the detection task) on the size of the attentional boost effect could be a promising avenue for future research. For instance, it has been speculated that the quantity of irrelevant items modulates whether an attentional boost effect is observed under incidental encoding instructions (see Dewald et al., 2013 for an extended discussion). In particular, the authors suggest that one may be more likely to observe an attentional boost effect under incidental encoding instructions when the number of irrelevant items is low and these items are repeated during the initial detection task. Following this line of reasoning, it is no surprise that there was no attentional boost effect under incidental encoding instructions in the present study, in which each picture was presented only once and the number of pictures was relatively large. However, as already mentioned in the introduction, participants may very well notice when a limited number of items is repeated several times during the detection task, which would undermine the incidental encoding instructions. Differently put, it is important to keep in mind that changing a methodological detail can have consequences reaching beyond the manipulation of this very detail.

In conclusion, the present study reveals two interesting findings. First, the encoding of perceptual information is enhanced at behaviorally relevant moments in time when encoding is intentional but not when encoding is incidental. Second, under incidental encoding conditions, still a relatively large amount of incoming information is stored in memory, independently of behavioral relevance and intention of memorization, indicating that perceptual long-term memory representations are formed as a natural product of perception.

## Data Availability Statement

The dataset presented in this study can be found in an online repository. A link can be found in the article.

## Ethics Statement

Ethical review and approval was not required for the study on human participants in accordance with the local participants and institutional requirements. The participants provided their written informed consent to participate in this study.

## Author Contributions

FH and CK developed the research idea, analyzed the data, and drafted the manuscript. FH designed the experiment. Both the authors contributed to the article and approved the submitted version.

### Conflict of Interest

The authors declare that the research was conducted in the absence of any commercial or financial relationships that could be construed as a potential conflict of interest.

## Footnotes

^1^Note, that Broitman and Swallow (2019) have demonstrated that the attentional boost effect can occur even when the stimuli are presented only once (Experiments 2A,B). However, this experimental setup was not combined with different encoding instructions (Experiment 1).

^2^https://www.dfg.de/foerderung/faq/geistes_sozialwissenschaften/
